# Integrated metabolomics and bioactivity analysis of *Cynoglossum lanceolatum* root extract as a natural inducer of resistance to rice bacterial blight

**DOI:** 10.3389/fgene.2026.1775858

**Published:** 2026-04-22

**Authors:** Aadil Mansoori, Madan Mohan, Subha Narayan Das, Rakesh Kumar, Anirudh Kumar

**Affiliations:** 1 Department of Botany, Indira Gandhi National Tribal University (IGNTU), Amarkantak, India; 2 Department of Life Sciences, Central University of Karnataka, kalaburagi, India; 3 Department of Botany, Central Tribal University of Andhra Pradesh, Vizianagaram, India

**Keywords:** antimicrobial, bacterial blight resistance, Cynoglossum lanceolatum crop protection, GC-MS, metabolites, phytochemicals, rice

## Abstract

**Background:**

Synthetic pesticides are widely used in agriculture to manage pests and reduce yield loss. Phytochemicals with antioxidant and antibacterial activities have great potential for treating plant diseases and reducing the use of synthetic chemicals. Identifying compounds from various plant species is crucial for their potential agricultural applications.

**Methods:**

In the present study, *Cynoglossum lanceolatum* was screened for potential antioxidant, antimicrobial, and bacterial blight protection abilities. Methanol and aqueous extracts of *C. Lanceolatum* root was tested for their polyphenol content, antioxidant potential, metabolomics and antimicrobial study.

**Results:**

Results revealed that methanol extract exhibited higher phytochemical content and antioxidant activity. FTIR examination of extracts identified functional groups such as OH, C-H, C=C, and C-N, indicating the presence of distinct metabolites. The GC-MS investigation indicated the existence of 59 metabolites, several of which had previously been described as antimicrobial agents. Furthermore, *in vitro* antibacterial studies confirmed the antimicrobial effect of methanol extract against *Xanthomonas oryzae* pv. *oryzae* (*Xoo*). Moreover, prediction of antimicrobial metabolites, particularly 7-hydroxy-4-methylcoumarin-3-acetic acid, was confirmed through molecular docking study with D-alanine-D-alanine ligase A (DdlA) and the peptide deformylase (PDF) protein of *Xoo.* Finally, the study evaluated the effectiveness of *C. lanceolatu*m root extract against bacterial blight disease, finding a significant reduction in *Xoo* lesions in pre-treatment and also showing their efficacy in post-treatment. Effect of extract was also observed in the photosynthetic status of rice by measuring chlorophyll A fluorescence.

**Conclusion:**

*C. lanceolatum* is a promising plant for its versatile role as an antioxidant, antimicrobial, and bacterial blight disease protection in rice.

## Graphical Abstract

**Figure:**
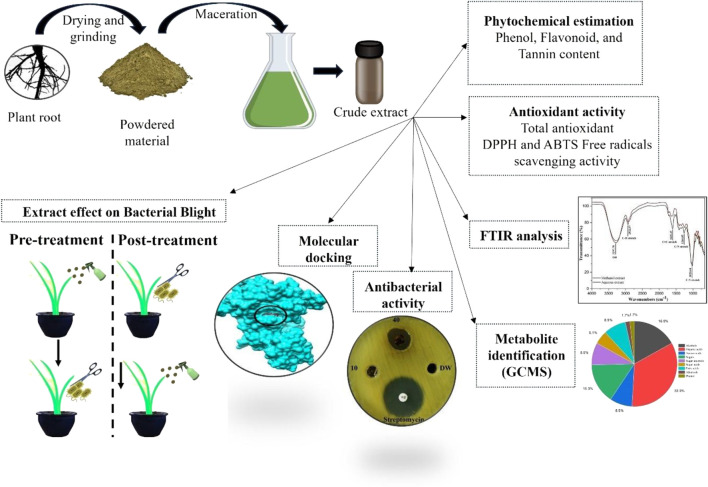

## 1 Introduction

Crops are frequently challenged by pathogens, which severely hamper their growth, production, and quality (Kumar et al., 2013; Richard et al., 2022). Farmers regularly apply synthetic chemicals to protect crops from these pests. The achievement of developmental objectives like food security, livelihoods, and economic growth has been made possible in large part by synthetic chemicals. However, inadequate and indiscriminate pesticide use results in detrimental health and environmental effects; as a result, several chemicals have been discontinued in agriculture (Anaduaka et al., 2023). Furthermore, the persistent use of synthetic chemicals enhances soil toxicity, harms beneficial microorganisms, hastens the decline of biodiversity, and promotes the emergence of resistant pathogens (Mansoori et al., 2022a; Shabana et al., 2017). Therefore, biological control offers an economical and sustainable means to address this serious problem. Plant-based products have become increasingly essential in the recent past for the development of better chemical pesticide substitutes for plant diseases. Plants containing bioactive chemicals such as phenolics, flavonoids, coumarins, alkaloids, tannins, and terpenoids have been used to treat plant, human, and animal infections (Anand et al., 2019; Valli et al., 2012; Mansoori et al., 2022b).

Rice (*Oryza sativa* L.) is the world’s primary food crop and the most valuable commodity that supports millions of people’s livelihoods and cultures (Shende, 2020; Kumar et al., 2020). As agriculture has advanced, infectious plant diseases have significantly affected crop productivity and profitability. Some infectious bacterial and fungal diseases, such as rice blast, sheath blight, and bacterial blight, have become increasingly critical factors influencing rice output and economic efficiency (Asibi et al., 2019; Doni et al., 2023). Bacterial blight (BB), caused by *Xanthomonas oryzae* pv. *oryzae* (*Xoo*), is considered the most severe and prevalent rice disease which can reduce yield by 20%–50%–100% during an epidemic (Kumar et al., 2013; Nazarov et al., 2020; Yang et al., 2020; Kumar et al., 2022). Chemical pesticides have almost entirely been used to control BB disease; however, their application has not proven effective because pathogenic races differ in their sensitivity to chemicals (Yasmin et al., 2017). Thus, as an alternative, plant-based bactericides might be a better option to overcome the negative chemical effects. Plant extracts act on pathogens directly as antimicrobial agents or trigger systemic resistance (Kagale et al., 2011; Singh et al., 2021). Hydrophobic and hydrophilic hydrocarbon skeleton functional groups of bioactive metabolites are critical for antibacterial action (Kha and Le, 2021). However, as resistance inducers, plant extract metabolites interact with cell receptors to produce a metabolic stimulus that leads to systemic resistance through a molecular defense response (Aziz et al., 2003). This defensive response attempts to halt the development of the invader and may result in SAR, making the plant less sensitive to further pathogen invasion (Henry et al., 2012).

Extracts of various plant species have been proven for their antibacterial potential and systemic resistance in hosts, consequently curbing the development of disease (Mansoori et al., 2020; Abo-Elyousr et al., 2020; Draz et al., 2019; Prasad et al., 2024). One such plant species is *Cynoglossum lanceolatum* Forssk. (Family: Boraginaceae), commonly known as Kamraaj (Ahirwar and Kujur, 2015). Traditionally, their roots have been used to treat eye problems and fever (Gupta and Gupta, 2017; Srivastava et al., 2012). Ahirwar and Kujur (2015) have also reported their effective role with *Murraya paniculata* for strength, vigor, and rheumatic pain. There are various reports about the medicinal properties of *C. lanceolatum*. However, there is limited research on phytochemicals and bioactive compounds from *C. lanceolatum* that serve as antagonists of phytopathogens or promote systemic resistance in rice. Currently, researchers are working on a variety of pathogen receptor proteins to understand the molecular mechanisms of the interactions between naturally occurring bioactive compounds and pathogen receptors. Therefore, the present study focused on quantitative phytochemical profiling, compositional analysis, *in vitro* and *in silico* antibacterial activity, and the effect of the extract on suppressing rice BB disease.

## 2 Materials and methods

### 2.1 Reagents

These studies employed only analytical grade chemicals and reagents. Chemicals such as gallic acid monohydrate, quercetin dihydrate, methanol, sodium carbonate, ammonium molybdate, 2,2-diphenyl-1-picrylhydrazyl (DPPH), 2,2′-azino-bis-3-ethylbenzothiazolin-6-sulfonic acid (ABTS), peptone, sucrose, calcium nitrate, agar-agar, ferrous sulfate, sodium phosphate monobasic, and l-ascorbic acid were procured from HiMedia (India). Aluminum chloride, sulfuric acid, and Folin–Ciocalteu reagent were purchased from Central Drug House (P) Ltd., India. Methionine was purchased from Sigma-Aldrich.

### 2.2 Plant materials and pathogen strain

Fresh *C. lanceolatum* plant materials were gathered near the Indira Gandhi National Tribal University (IGNTU) campus in Amarkantak, Madhya Pradesh (MP), India. Authentication of the plant’s taxonomic identity was carried out by an IGNTU taxonomist from the Departments of Botany and Environmental Sciences. The most susceptible genotype of rice Taichung Native 1 (TN 1) and a highly virulent strain of *Xoo* (IXO-20) were obtained from the ICAR-Indian Institute of Rice Research (IIRR) in Hyderabad, India.

### 2.3 Estimation of phytochemicals

#### 2.3.1 Phenol content

Phenol content in crude root extract was calculated by modifying the Folin-Ciocalteu method (Singleton et al., 1999). 0.3 mL of supernatant was combined with 2 mL of Folin-Ciocalteu reagent (10% w/v), and the resulting mixture was then incubated for 5 min. Next, 2 mL of 7.5% sodium carbonate was added to the entire mixture, and it was vortexed for 2 minutes. After 45 min of incubation, absorbance was finally measured at λmax = 750 nm. Gallic acid in the range of 20–100 μg/mL was used to make a standard calibration curve. The phenol content was expressed as mg of gallic acid equivalents (mg GAE g^-1^).

#### 2.3.2 Flavonoid content

Flavonoid content was estimated through the aluminum chloride (AlCl_3_) colorimetric method (Chang et al., 2002). The reaction mixture was prepared by mixing 10% AlCl_3_ (0.1 mL) with 0.5 mL of supernatant. DDW was added to the mixture to bring the final volume up to 2 mL. Finally, absorbance at λmax = 415 nm was measured following a 2-min vortex and a 30-min dark incubation period. A standard curve of quercetin was prepared (20–100 μg/mL), and the flavonoid content in the extract was expressed as mg of quercetin equivalents per Gram of dry weight (mg QE g^-1^).

#### 2.3.3 Tannin content

Tannins were determined using the Folin-Ciocalteu method (Singh et al., 2012). Root extract of 0.1 mL was added to 0.5 mL of Folin-Ciocalteu phenol reagent (10%) and 1 mL of 35% sodium carbonate solution. The volume of the reaction mixture was made up to 2 mL with distilled water, followed by incubation for 30 min. Tannic acid reference standard solutions (20–100 μg/mL) were prepared in the same manner as described above. The absorbances of the test and standard solutions were measured at a maximum wavelength of 725 nm. The tannin concentration was calculated in milligrams of tannic acid equivalent per Gram of extract (mg TAE g^-1^).

### 2.4 Determination of antioxidant activity

#### 2.4.1 Total antioxidant activity (TAA)

TAA of the extract was determined using the standard procedure with a few modifications (Govindarajan et al., 2003; Subhasree et al., 2009). In short, 0.2 mL of the extract was added to 1.8 mL of reagent containing 0.6 M H_2_SO_4_, 28 mM Na_3_PO_4_, and 4 mM (NH_4_)_6_Mo_7_O_24_). The reaction mixture was incubated in a water bath at 90 °C for 90 min. After cooling to room temperature (RT), the absorbance of the sample was read at λmax = 695 nm using a spectrophotometer. Ascorbic acid was used as a standard (20–100 μg/mL), and TAA results were represented as mg of ascorbic acid equivalent per Gram of plant extract.

#### 2.4.2 2,2-Diphenyl-1-Picrylhydrazyl (DPPH) radical scavenging activity

DPPH assays were used to assess the extract’s free radical scavenging activity (Bursal and Gülçin, 2011). The solution was made by dissolving 4.0 mg of DPPH in 100 mL of methanol, and absorbance was measured using a spectrophotometer. This solution was mixed with 300 µL of either a standard (Ascorbic acid) or an extract at different concentrations (20–220 μg/mL). Following shaking, the reaction mixture was incubated at RT for 15 min in the dark. At λ max = 515 nm, the maximum absorbance was measured. Using the equation below, the scavenging activity was calculated in terms of the DPPH radical scavenged.

$$\text{Scavenging effect }\%=\left(\text{control absorbance}-\text{sample absorbance}\right)\times 100/\text{control absorbance}.$$

#### 2.4.3 2,2′-Azino-Bis-3-Ethylbenzothiazoline-6-Sulfonic Acid (ABTS) radical scavenging activity

The crude plant extract’s ability to scavenge ABTS radicals was evaluated using a standard approach (Re et al., 1999). The decolorization of ABTS^•+^ radicals via the electron-donating activity of plant extract antioxidants forms a component of the reaction process. DDW was used to make a 7.0 mM ABTS solution, which was then mixed in a 1:1 ratio with a 2.45 mM K_2_S_2_O_8_ solution. Following that, a 24- to 48-h dark incubation at RT was undertaken. After diluting the ABTS solution with methanol, the absorbance was adjusted to 0.700 0.02 at max = 745 nm. A 2.5 mL solution was mixed with extract/standard concentrations ranging from 20 to 220 μg/mL (300 µL). After shaking, the whole reaction mixture was incubated at RT for 15 min in the dark. Finally, absorbance at λmax = 745 nm was determined. The scavenging activity was determined in terms of ABTS^•+^ percent inhibition using the same equation as the DPPH radical scavenging activity.

#### 2.4.4 Fourier-transform infrared (FTIR) spectroscopy analysis

FTIR spectroscopy was used to determine the functional groups in methanol and aqueous extract of *C. lanceolatum* root using a Thermo Nicolet iS5 instrument (Thermo Scientific, Madison, WT, United States) equipped with KBr windows. The readings were carried out at RT. The range of the measured spectra was between 4,000 and 650 cm^−1^. At last, the OriginPro 2021 software was used for peak selection and results analysis.

#### 2.4.5 Metabolites identification through gas chromatography-mass spectrometry (GC-MS)

Gas chromatography-mass spectrometry (GC-MS) was employed to identify the presence of metabolites, as described elsewhere (Sharma et al., 2021; Kilambi et al., 2021; Roessner et al., 2000). A metabolite fraction was formed by combining 50 mg of dry powder with 0.95 mL of 100% chilled methanol containing ribitol as an internal standard, followed by a 15-min incubation at 70 °C with constant shaking. After incubation, 0.95 mL of chilled water was added, and centrifugation was carried out at 2,500 g for 15 min at 4 °C. The supernatant was transferred to a new centrifuge tube and dried for 120 min. Derivatization of the obtained residue was accomplished by incubating it in methoxamine (65 L) containing pyridine at 37 °C for 90 min after incubating it in N-methyl-N-(trimethylsilyl) trifluoroacetamine and FAME mix (20 µL) at 37 °C for 30 min. The derivatized sample was analysed using the LECO-PEGASUS GCXGC-TOF-MS system (LECO Corporation, USA), which was set up with a 30-m Rxi-5ms column having 0.25-mm internal diameter and 0.25-m film thickness (Restek, USA). The injection temperature, interference temperature, and ion temperature were all set to 240 °C, 225 °C, and 200 °C, respectively. For sample chromatographic separation, the following procedures were recommended: 5 min of isothermal heating (70 °C), followed by a 5 °C min^-1^ oven temperature ramp to 290 °C and 5 min of heating at 290 °C. Helium gas was used as the carrier, and the flow rate was set to 1.4 mL/min. Finally, 1 µL of the sample was supplied in splitless mode for analysis, with the scan mass range adjusted to 70 to 600 at two scans/s.

Metabolite identification was executed as per standard protocol (Sharma et al., 2021). Data files were created as netCDF by the ChromaTOF programme 299 4.50.8.0 chromatographic version (LECO Corporation, USA) and analysed with MetAlign 3.0 software (www.metalign.nl) (Lommen and Kools, 2012). Several parameters were examined, including the signal-to-noise ratio ≥2, baseline correction, noise estimation, and mass peak identification (ion-wise mass alignment). MSClust analysis was used to reduce data and extract compound mass (Tikunov et al., 2012). Finally, the files were transferred to the NIST MS Search v2.2 program, which used the NIST (National Institute of Standards and Technology) library and the GOLM metabolome database library to identify chemical compounds (Hummel et al., 2010). All metabolites were determined by normalization with ribitol as an internal standard.

#### 2.4.6 Antibacterial ctivity against *Xanthomonas oryzae* pv. *oryzae*

The antibacterial activities of methanol and aqueous extract of *C. lanceolatum* root extracts were assessed using the agar well diffusion method (Daoud et al., 2019; Gonelimali et al., 2018). Fresh *Xoo* suspension (100 µL) was pipetted and placed into a sterile Petri plate containing modified Wokimoto’s medium. After solidification, wells were drilled into agar plates containing inoculums with a sterile cork borer (6 mm diameter). Next, 100 µL of each extract (10 and 40 mg/mL) was added to the corresponding wells. The plates were placed in the refrigerator for 30 min to diffuse the extracts into the agar properly, then incubated at 28 °C for 48 h. Streptomycin and milli-Q water were used as positive and negative controls, respectively. Finally, antibacterial activity was assessed as the diameter of the bacterial zone of inhibition in centimeters.

#### 2.4.7 Prediction of metabolite’s binding affinity through molecular docking analysis

Docking analysis was performed on the 41 metabolites from the GC-MS data. These chemicals were targeted to inhibit D-alanine D-alanine ligase A (DdlA) (PDB ID-4L1K) and Peptide deformylase (PDF) (PDB ID-5E5D). The three-dimensional structure of the target protein was retrieved from the Protein Data Bank (https://www.rcsb.org/). BIOVIA Discovery Studio was used to eliminate heteroatoms including water and other molecules of protein. The missing amino acid residue in the protein was repaired using Swiss-PdbViewer (v4.1). Energy minimization and active site prediction were executed in the UCSF Chimaera and the Computed Atlas of Surface Topography of Protein (CASTp) web services, respectively. Following protein preparation, each ligand’s 3D structure was downloaded in SDF format from PubChem (https://pubchem.ncbi.nlm.nih.gov/) and imported into PyRx for energy minimization and PDBqt conversion. Grid settings were adjusted for both proteins to assist them navigate the active site and bind to the ligand properly. The centre grid box values for DdlA were x = 1.69, y = 10.80, and z = 32.03, while for PDF they were x = 12.27, y = −14.59, and z = −7.91. Grid points in the x, y, and z dimensions for both receptor proteins were set to 20.39, 25.0, and 28.29 (DdlA) and 25.0, 20.05, and 16.51 (PDF), respectively. AutoDockVina implemented in PyRx software was to conduct virtual screening for target protein active sites (Trott and Olson, 2010). Finally, the binding energy table was retrieved in Excel format from the PyRx software. Compounds with high binding affinities were evaluated for 3D structure and bond formation with participating amino acids using BIOVIA Discovery Studio 2016.

#### 2.4.8 Effect of *C. lanceolatum* root extract on bacterial blight disease

**FIGURE 1:**
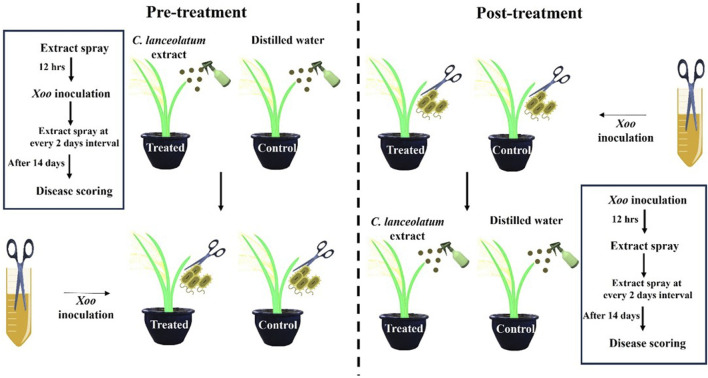
Experimental scheme for BB protection. Extract: rice sprayed with an aqueous extract of *C. lanceolatum* root. Control: rice sprayed with distilled water. Rice plants were sprayed with extract and distilled water at 12 h before and after *Xoo* inoculation, followed by regular spray every 2 days of intervals. The disease was scored on the 14th day after inoculation.

Rice plants were grown naturally in earthen pots at the IGNTU nursery until they reached the maximum booting stage. Following that, healthy plant pots were transported to the laboratory for a 10-day disease monitoring period. The experiment was done on a 60-day-old rice plant. To prepare the *Xoo* inoculum, IXO-20 strains were cultured in a modified Wakamoto’s medium and incubated at 28 °C. The cells were then collected and centrifuged to remove the exopolysaccharides. The cells were then resuspended in DDW, producing over 10^9^ CFU per ml. Fully grown leaves were sprayed with extract (2 mg/ml) (Figure 1). After 12 h, the leaves were infected with *Xoo* by the leaf clipping method, which includes dipping scissors into the *Xoo* suspension. Leaves treated with DDW were used as a negative control. After extract treatment and pathogen inoculation, leaf samples were collected for disease scoring.

### 2.5 Statistical analysis

Experiments were conducted in triplicate and results were expressed as mean ± standard deviation (SD). The sample’s data calculation was conducted in Microsoft Excel 2016. The Student’s t-test was employed to determine the significant difference between the two samples or controls. Statistical analysis and graph creation were performed using GraphPad Prism 9.3.3 and OriginPro 2021 software.

## 3 Results and discussion

### 3.1 *C. lanceolatum* root extract exhibited high content of phenol, flavonoids, and tannin

**FIGURE 2:**
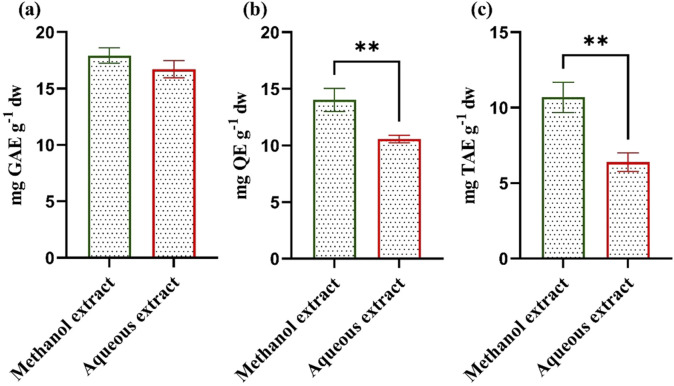
Phytochemicals estimation of *C. lanceolatum* methanol and aqueous extract. **(a)** Total phenolic content, **(b)** total flavonoid content, **(c)** Total tannin content. Phenol and tannin content were calculated from the calibration curve of gallic acid, while quercetin calibration curve was used for flavonoids calculation. Each value is mean of three biological replicates.

For phytochemicals investigation, phenol, flavonoids, and tannin content were estimated in the methanol and aqueous extracts of *C. lanceolatum* root (Figure 2). Phenolic content was calculated using the gallic acid calibration curve equation (y = 0.0079x - 0.0005, R^2^ = 0.99), while flavonoids and tannin content were estimated from the equations obtained from the standard quercetin (0.0056x - 0.042, R^2^ = 0.97) and tannic acid (0.0041x + 0.0283, R^2^ = 0.99) curves, respectively (Supplementary Figure S1). The methanol (17.93 ± 0.55 mg GAE g^-1^dw) and aqueous (16.7 ± 0.62 GAE g^-1^dw) extracts demonstrated almost similar phenol content. Conversely, the flavonoid content of the methanol extract (14.01 ± 0.84 mg QE g^-1^dw) was significantly greater (p < 0.01) than that of the aqueous extract (10.56 ± 0.27 mg QE g^-1^dw). With regards to mg of TAE g^-1^dw, the tannin content was also exhibited to be greater in methanol (10.69 ± 0.82) than in aqueous extract (6.39 ± 0.50). Phenols and flavonoids are known to contain numerous bioactivities such as anti-cancer, anti-inflammatory, anti-allergic, tumor inhibitory, and antibacterial attributes (Dubey et al., 2023; Liu et al., 2023). Plants with a high phenolic content can potentially be used for many medicinal purposes in treating chronic illnesses caused by microbial infections through the mechanisms of permeabilization and instability of the plasma membrane or the inhibition of enzymes outside of cells (Górniak et al., 2019; Takó et al., 2020). Likewise, different antibacterial mechanisms of plant flavonoids were reported, including their target on synthase enzymes for cell walls, nucleic acid, and bacterial respiration (Górniak et al., 2019; Xie et al., 2014). Tannin, a typical phenol, has no clear understanding of the factors that explain its antimicrobial activity. However, the observable activity of a substance may be attributed to the presence of free phenolic hydroxyl groups, which can influence its enzymatic activity through covalent or non-covalent linking (Villanueva et al., 2023). The presence of phenolics and flavonoids in *C. lanceolatum* makes it an effective antibacterial agent since it has previously shown microbial inhibitory effects (Dzotam et al., 2018; Jarial et al., 2018). As a consequence, the results show that *C. lanceolatum* would be of great interest for further research.

### 3.2 *C. lanceolatum* root extract has capacity to combat free radical mediated damage

**FIGURE 3:**
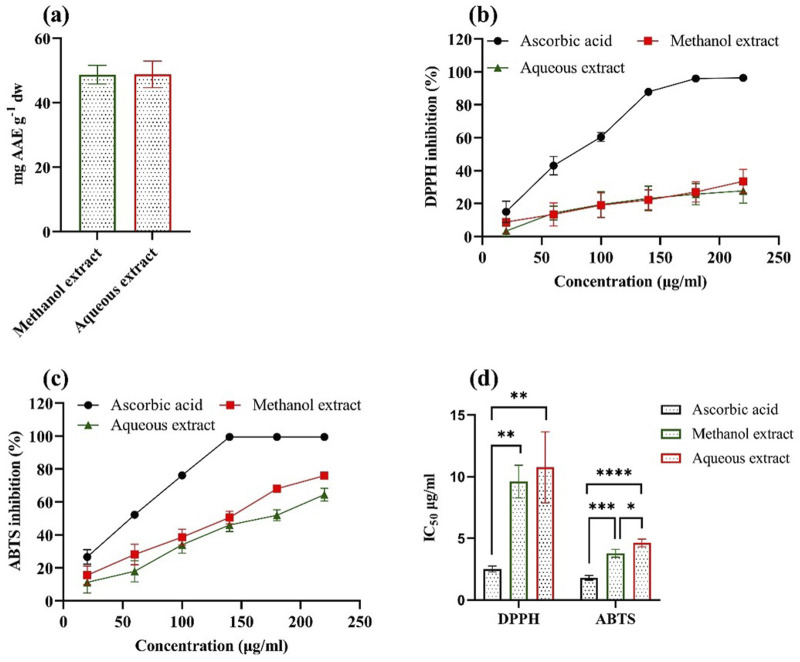
Antioxidant activity of *C. lanceolatum* methanol and aqueous extract. **(a)** Total antioxidant activity, **(b)** DPPH, and **(c)** ABTS radical scavenging activity (20–220 μg/mL), **(d)** IC_50_ values of DPPH and ABTS radical scavenging activity. Each value is average of three replicates n = 3 ± SD. Total antioxidant activity was calculated from the calibration curve of ascorbic acid. Asterisk represent significant difference between each other (*p < 0.05; **p < 0.01; ***p < 0.001; ****p < 0.0001; One way ANOVA).

Antioxidant activity in the extract was evaluated using phosphomolebdenum method. Phosphomolybdenum assay explains the total antioxidant activity of sample which was calculated using equation (y = 0.0035x + 0.0951, R^2^ = 0.99) (Supplementary Figure S2). The results suggested that methanol extract (14.01 ± 0.84 AAE g^-1^dw) has higher TAA in compare to aqueous extract (10.56 ± 0.27 AAE g^-1^dw) (Figure 3a). Antioxidant activity of compounds has found to be associated with free radical scavenging activity; thus, extract capacity was further accessed for their reducing ability to synthetic oxidants (DPPH&ABTS). Figure 3b depicts concentration-dependent (20–220 μg/mL) changes in DPPH radical scavenging capability of *C. lanceolatum* extract. For DPPH inhibition, both methanol and aqueous extract has shown almost equal radical scavenging potential with IC_50_ value of 9.60 ± 1.08 μg/mL and 10.75 ± 2.35 μg/mL respectively. However, in compare to extract, the standard (ascorbic acid) scavenging activity was found to be higher. Further, in the case of ABTS inhibition, methanol extract exhibited slightly higher inhibition than aqueous extract in each concentration. Moreover, IC_50_ values of methanol extract (3.79 ± 0.25 μg/mL) was significant lower compare to aqueous extract (4.62 ± 0.25 μg/mL) which indicates the potential ABTS radical scavenging ability (Figures 3c,d). The above finding supports the significant contribution of flavonoids and phenols to the overall antioxidant activity of extract linked to phosphomolybdate scavenging. Several studies have also shown a correlation between total phenol and antioxidants (Aryal et al., 2019; Paixão et al., 2007). The DPPH and ABTS test is an appropriate and frequently used approach for assessing the free radical scavenging capabilities of the sample (Xiao F et al., 2020; Mansoori et al., 2022a). Phenolic substances contain hydroxyl groups that are accountable for eliminating free radical’s actions owing to their redox attributes (Olszowy, 2019). Research suggested that the natural antioxidants produced by plants have been described as antibacterial agent with plausible mechanisms of microbial enzyme inhibition, and protein regulation.

### 3.3 FT-IR analysis of *C. lanceolatum* root extract leads to identification of several functional groups

**FIGURE 4:**
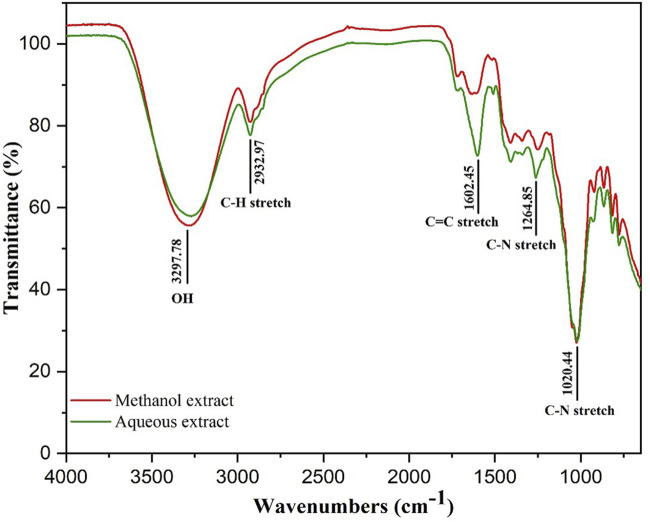
FT-IR spectrum of methanol and aqueous extract of *C. lanceolatum* root and their probable functional groups with wavenumbers (4000–650 cm^-1^).

The FT-IR analysis identified several functional groups based on multiple peaks present in the methanol and aqueous extract. FTIR executes a distinct qualitative examination and presents a chemical signature, as no two bioactive chemicals would have identical spectra (Easmin et al., 2017). Both methanol and aqueous extract exhibited a comparable infrared spectrum (IR) profile, which can be attributed to the presence of polar molecules in the extracts (Figure 4). The spectra of both extracts displayed a wide peak of OH at 3297.78 cm^-1^, which signifies the stretching of the polymeric hydroxyl group, a distinctive feature of polyphenolic chemicals (Wongsa et al., 2022). The occurrence of C-H stretching at 2932.97 cm^-1^ suggests the presence of compounds associated with aldehyde and alkene. Additional peaks at 1602.45 and 1664.85 cm^-1^ show the presence of C=C and C-N functional group. Finally, the identification of OH, C-H, C=C, and C-N in methanol and aqueous extract of *C. lanceolatum* indicates the most probable nature of chemicals such as phenolics, amines, alkanes, and aldehydes, which could be responsible for the antimicrobial properties of the extract (Mansoori et al., 2020).

### 3.4 GC-MS based metabolomics analysis revealed potential antimicrobial metabolites

**FIGURE 5:**
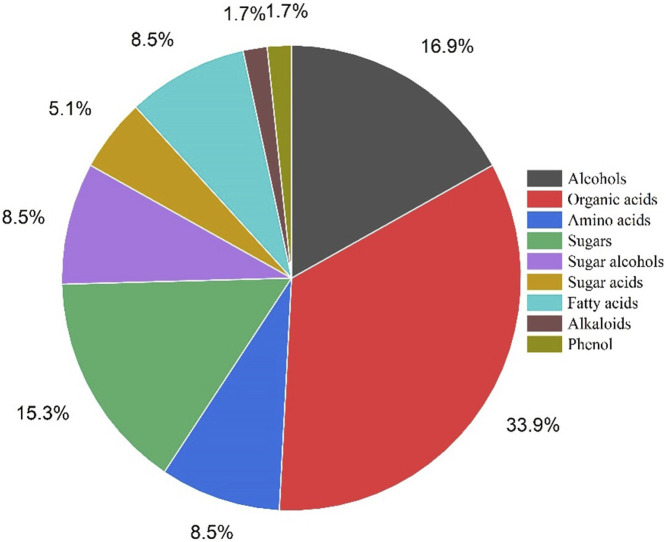
Metabolite groups with percentage in *C. lanceolatum* root extract.

Only FTIR analysis is insufficient to confirm the existence of distinct classes of chemicals in the extract. Consequently, the methanol extract of *C. lanceolatum* root was proceeded to GC-MS for metabolite detection. A total of 59 metabolites have been identified and grouped into separate classes, comprising alcohols (16.9%), organic acids (33.9%), amino acids (8.9%), sugars (15.3%), sugar alcohols (8.5%), sugar acids (5.1%), fatty acids (8.5%), as illustrated in Figure 5. However, alkaloid and phenolics comprises each 1.7% among other groups. Each group of metabolites was assessed in terms of relative level concerning internal standard (ribitol) (Table 1). Natural plant substances belonging to various kinds of secondary metabolites are renowned for their great involvement in pathogen suppression (Babosha, 2004). Several metabolites identified in the extract have been previously reported for their antibacterial properties against different pathogens (Supplementary Table S1). Presence of echinatine, a pyrrolizidine alkaloids confirms their specificity as it is found majorly in genera *Cynoglossum* (El-Shazly and Wink, 2014; Saini, 2016).

**TABLE 1:** List of metabolites and their relative level identified methanol extract of *C. lanceolatum* root extract.

Metabolites^a^	Groups	CL1^b^	CL2	CL3	CL4	CL5	CL6	CL mean relative level
Pyruvic acid	Organic acids	0.17977	0.25783	0.23843	0.22183	0.21296	0.18656	0.22 ± 0.03
Lactic acid		0.86473	0.52159	0.75251	0.98594	0.82201	0.85258	0.80 ± 0.16
Glycolic acid		10.6016	9.02777	7.50976	10.7091	8.21338	8.92612	9.16 ± 1.28
3-Hydroxypropionic acid		1.80536	1.53293	1.63313	2.16752	1.74962	1.89129	1.80 ± 0.22
4-Hydroxybutanoic acid		0.35509	0.32295	0.40537	0.45891	0.39815	0.44351	0.40 ± 0.05
Phosphoric acid		13.2987	13.6197	13.48	15.57	15.1032	12.8149	13.98 ± 1.09
Succinic acid		1.91627	1.97465	1.85167	2.12752	1.73883	1.70815	1.89 ± 0.16
Glyceric acid		1.91675	1.76365	1.58209	1.86271	1.54971	1.50283	1.70 ± 0.17
2-Furanglycolic acid		0.17394	0.20847	0.17898	0.22493	0.19477	0.19335	0.20 ± 0.02
4-N-Methylaminobutyric acid		0.06394	0.07901	0.08758	0.06452	0.05767	0.05762	0.07 ± 0.01
2-Deoxytetronic acid		0.12001	0.10974	0.11256	0.12551	0.08841	0.10418	0.11 ± 0.01
2,4-Hexadienedioic acid		1.02144	1.3053	1.15376	1.14305	0.93233	0.91198	1.08 ± 0.15
Malic acid		12.2861	16.5008	15.0645	12.1216	13.9829	11.8694	13.64 ± 1.88
L-Threonic acid		0.35419	0.36807	0.39631	0.32192	0.31662	0.28613	0.34 ± 0.04
3,4,5-Trihydroxypentanoic acid		0.20684	0.35022	0.27288	0.20559	0.24887	0.17284	0.24 ± 0.06
Citric acid		0.07574	0.21743	0.20967	0.064	0.17656	0.14471	0.15 ± 0.07
3,4-Dihydroxyphenylacetic acid		0.09643	0.10924	0.09966	0.089	0.09353	0.07811	0.09 ± 0.01
2-Butenedioic acid/Fumaric acid		0.1848	0.21952	0.21653	0.16881	0.2013	0.26475	0.21 ± 0.03
Ribonic acid, gamma-lactone		0.16256	0.15338	0.15478	0.16057	0.13867	0.13939	0.15 ± 0.01
7-Hydroxy-4-methylcoumarin-3-acetic acid		0.01691	0.04573	0.03686	0.02304	0.02082	0.01612	0.03 ± 0.01
Ethylene glycol	Alcohols	0.1618	0.16012	0.21821	0.2075	0.16606	0.17904	0.18 ± 0.02
2,3-Butanediol		0.09574	0.10591	0.10974	0.11451	0.12681	0.10363	0.11 ± 0.01
Furfuryl alcohol		0.81004	0.92401	0.85546	0.77299	0.69263	0.79217	0.81 ± 0.08
2-Heptanol		0.00034	0.00049	0.00023	0.00024	0.00016	0.00016	0.0003 ± 0.00
5-Hydroxymethylfurfural		1.36458	2.45285	1.71765	1.82208	1.97067	1.63024	1.83 ± 0.37
Pyridine, 2-hydroxy		0.79072	0.66413	0.76119	0.70547	0.74301	0.64261	0.72 ± 0.06
1,3-Butanediol		2.41981	2.54355	2.23613	2.80766	2.37011	2.65792	2.51 ± 0.21
Glycerol		10.8555	9.21808	7.94262	11.3246	6.93056	9.71261	9.33 ± 1.68
1,2,3-Butanetriol		0.07956	0.07867	0.09916	0.09862	0.07718	0.08324	0.09 ± 0.01
1,2,4-Butanetriol		0.15646	0.21947	0.20036	0.17549	0.17891	0.15296	0.18 ± 0.03
meso-Erythritol	Sugar alcohols	1.71221	1.7459	1.77521	1.84719	1.93668	1.95213	1.83 ± 0.10
D-Erythro-Pentitol, 2-deoxy		0.29732	0.38508	0.32791	0.22338	0.33748	0.25601	0.30 ± 0.06
1,5-Anhydroglucitol		6.04016	7.30762	6.96334	6.26984	5.78471	5.5262	6.32 ± 0.69
Myo-Inositol		2.41466	2.05603	2.45234	2.38589	2.71215	1.97165	2.33 ± 0.27
Sorbitol		3.36388	3.00327	4.61722	3.86321	3.56378	2.38997	3.47 ± 0.76
D-Ribo-Hexonic acid	Sugar acids	1.33843	1.79203	1.67183	1.48811	1.58082	1.31046	1.53 ± 0.19
D-galacturonic acid		0.54654	0.447	0.65831	0.6	0.39492	0.41342	0.51 ± 0.11
D-Gluconic acid		0.61283	0.73516	0.69218	0.65973	0.74334	0.38275	0.64 ± 0.13
4-Aminobutanoic acid/GABA		0.1406	0.10982	0.11455	0.14622	0.08372	0.15517	0.13 ± 0.03
L-5-Oxoproline/Pyroglutamic acid	Amino acids	1.80801	1.67332	1.73511	1.83843	1.32488	1.79768	1.70 ± 0.19
2-Aminobenzoxazole		0.19875	0.17431	0.15612	0.15764	0.19279	0.18964	0.18 ± 0.02
Dihydroxyphenylalanine		9.20749	9.28098	10.6487	10.7485	11.8589	9.58574	10.22 ± 1.04
trans-4-hydroxy-L-proline		1.0423	1.29374	1.20208	1.11392	1.03514	0.9996	1.11 ± 0.11
Caffeic acid	Phenol	0.08692	0.09765	0.1261	0.13176	0.13452	0.10865	0.11 ± 0.02
Echinatine	Alkaloids	3.30248	4.80411	4.69802	4.0723	4.40447	3.38126	4.11 ± 0.65
Palmitic acid	Fatty acids	1.22872	1.06231	1.18275	1.44631	1.39235	1.20118	1.25 ± 0.14
Linoleic acid		0.22352	0.19933	0.20529	0.20789	0.21758	0.20363	0.21 ± 0.09
Oleic acid		0.16644	0.14416	0.13463	0.14739	0.15776	0.12553	0.15 ± 0.01
Elaidic acid		0.14914	0.09221	0.10137	0.09914	0.08968	0.07916	0.10 ± 0.02
Stearic acid		1.05641	0.72028	0.81247	0.99442	1.04142	0.91763	0.92 ± 0.13
Erythrono-1,4-lactone	Sugars	0.4357	0.44713	0.41533	0.5129	0.33181	0.43031	0.43 ± 0.06
Threose		0.04749	0.05559	0.04775	0.05507	0.0442	0.04292	0.05 ± 0.05
Lyxose		1.78139	2.17236	2.00977	2.05579	1.81807	1.77963	1.94 ± 0.17
Fructose		296.597	313.417	369.046	346.696	317.341	279.554	320.44 ± 32.71
Mannose		161.958	144.547	160.691	138.678	139.282	146.854	148.67 ± 10.29
d-Galactose		56.7811	62.8046	75.4954	66.5726	60.6754	50.6663	62.17 ± 8.50
D-glucose		38.241	48.1893	32.2171	41.0354	42.1654	43.9753	40.97 ± 5.42
Maltose		0.94326	0.97805	0.89637	0.71722	0.67894	0.92146	0.86 ± 0.13
Sucrose		5.07045	4.52894	4.40738	3.40071	3.16241	2.80216	3.90 ± 0.90

^a^ Metabolites and their relative levels are in six biological replicates. The method of identification is RI/SD/MSMS, based on the RI, of the NIST, and GOLM, database. Metabolites are categorized based on the functional group present.

^b^ CL, with different numbers represents *C. lanceolatum*replication.

### 3.5 *C. lanceolatum* extract exhibited antibacterial activity against *Xoo*

**FIGURE 6:**
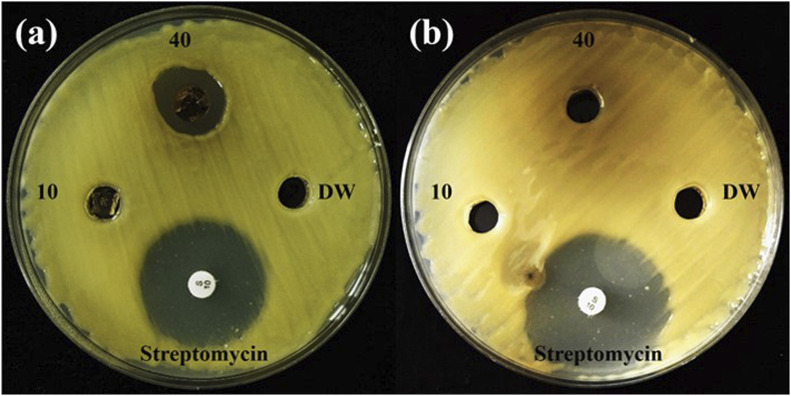
Antimicrobial activity of methanol **(a)** and aqueous extract **(b)** of *C. lanceolatum*. 10 and 40 are concentration in mg/ml. Streptomycin and distilled water (DW) were used as the positive and negative control, respectively. Both methanol and aqueous extract showed a zone of inhibition only at 40 mg/mL concentration.

In the present study, it has been observed that only methanol extracts exhibited antagonist activity at a concentration of 40 mg/mL. Methanol extract has shown the highest zone of inhibition (17.83 ± 0.76 mm). Notably, no inhibitory activity was perceived with the aqueous extract (Figure 6). However, the positive control, streptomycin, exhibited a zone of inhibition of 34.70 ± 0.45 mm. It is commonly acknowledged that the antibacterial capacity of plant extracts is regulated by the constituents of their secondary metabolites. Thus, the extract has found different metabolites in GC-MS analysis, and some of those have been reported as antimicrobial agents previously. Specifically, caffeic acid and its derivates, a phenolic compound, have been investigated tremendously against different microorganisms for antibacterial activity (Khan et al., 2021; Lima et al., 2016; Ma et al., 2018; Merlani et al., 2019). Hence, their presence in methanol extract can correlate with their activity against *Xoo*.

### 3.6 Prediction of metabolites binding against *Xoo* receptors

**FIGURE 7:**
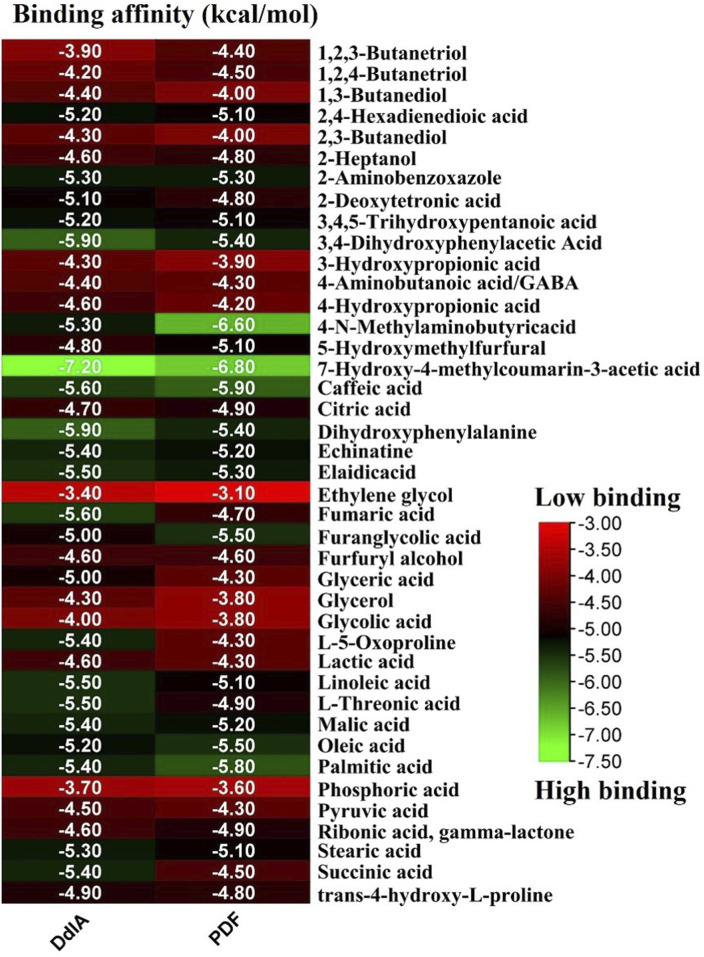
Binding affinity of various metabolites for DdlA and PDF protein of *Xoo*. The colors green and red signify strong and weak binding affinity, respectively.

**FIGURE 8:**
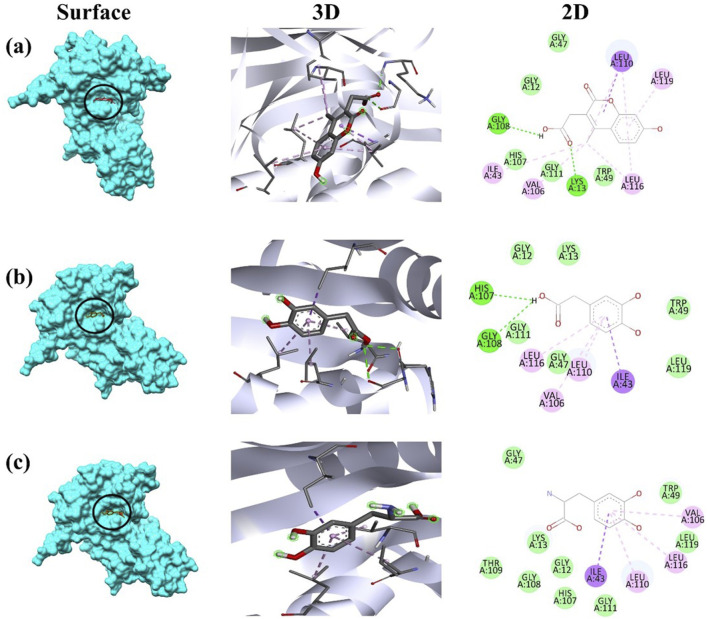
Molecular docking interaction and different bond formation of 7-hydroxy-4-methylcoumarin-3-acetic acid **(a)** glucose-1-phosphate, **(b)** 3,4-dihydroxyphenylacetic acid, and **(c)** dihydroxyphenylalanine with DdlA protein of *Xoo.*

**FIGURE 9:**
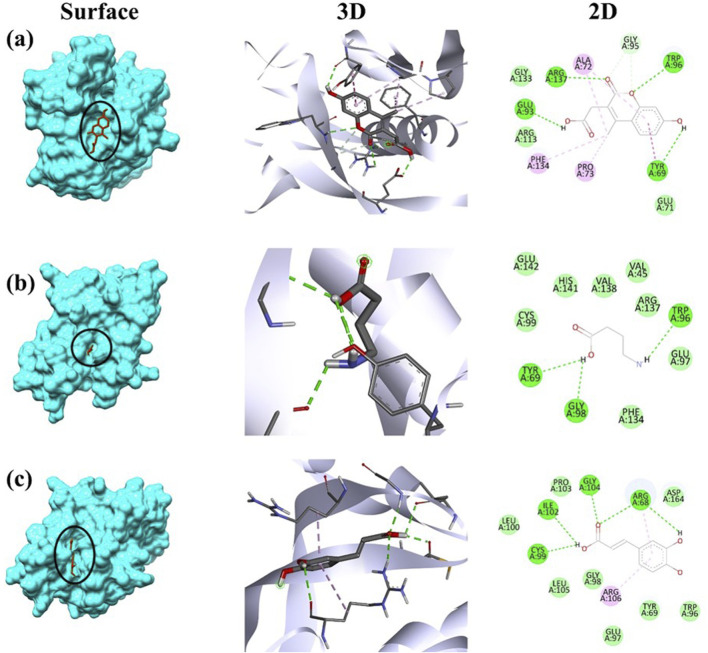
Molecular docking interaction of 7-hydroxy-4-methylcoumarin-3-acetic acid **(a)**, 4-N-methylaminobutyricacid **(b)**, and caffeic acid **(c)** with PDF protein of *Xoo*.

To investigate binding affinity, 41 compounds (excluding sugar compounds) were docked to DdlA and the PDF of *Xoo*. Virtual screening approaches are frequently and widely utilized to identify novel ligands for protein structures. To investigate binding affinity, 41 compounds (excluding sugar compounds) were docked to DdlA and the PDF of *Xoo*. Virtual screening approaches are frequently and widely utilized to identify novel ligands for protein structures. The binding affinity of metabolites with DdlA ranged from −3.4 to −7.2 kcal/mol (Figure 7). 7-Hydroxy-4-methylcoumarin-3-acetic acid demonstrated the highest binding affinity (−7.2 kcal/mol). Both 3,4-dihydroxyphenylacetic acid and dihydroxyphenylalanine had a binding affinity of −5.9 kcal/mol with the DdlA protein. In PDF, 7-Hydroxy-4-methylcoumarin-3-acetic acid had the highest binding affinity (−6.8 kcal/mol), followed by 4-N-Methylaminobutyricacid (−6.6 kcal/mol) and Caffeic acid (−5.9 kcal/mol). Additionally, structural interaction studies for bond formation were visualized in Discovery Studio. 7-Hydroxy-4-methylcoumarin-3-acetic acid formed two hydrogen bonds with the Lys13 and Gly108 residues of DdlA proteins (Figure 8a). 3,4-dihydroxyphenylacetic acid also established two hydrogen bonds with His:107 and Gly:108 (Figure 8b). However, no hydrogen bond was established between the dihydroxyphenylalanine and DdlA residues (Figure 8c). PDF and 7-Hydroxy-4-methylcoumarin-3-acetic acid formed six hydrogen bonds with distinct amino acids, including Tyr:69, Glu:93, Gly:95 (Two bonds), Trp:96, and Arg:137 (Figure 9a). However, 4-N-Methylaminobutyric acid was capable of forming three hydrogen bonds with PDF (Figure 9b). Caffeic acid and PDF interaction resulted in five hydrogen bonds with Cys:99, Ile:102, and Gly:104, and Arg:68 (Two bonds) (Figure 9c). Other hydrophobic bonds of both the proteins with metabolites are depicted in Supplementary Table S2. DdlA is bacterial cell wall synthesis essential enzyme. Likewise, PDF catalyze the removal of N-formyl group from N-terminal methionine during translation. Both proteins play an essential role in *Xoo*, which make them good target to formulate new inhibitoragainst both proteins. 7-Hydroxy-4-methylcoumarin-3-acetic acid found to be excellent inhibitor with higher binding affinity. In general it has been noticed that the compound having basic coumarin structure is proved as an efficient antimicrobial agents (Alshibl et al., 2020; Sahoo et al., 2021). The antibacterial activities of coumarin derivatives have shown better activity against Gram-positive bacteria such as *Bacillus subtilis* and *Staphylococcus aureus* than Gram-negative bacteria particularly against *Escherichia coli* and *Pseudomonas aeruginosa* (Lin et al., 2012). Structural modifications of coumarin further enhances the biological activities, for examples, derivatives containing thiosemicarbazide and 4-thiazolidinone groups have shown strong antifungal activity against foodborne fungi such as *Aspergillus flavus*, *Aspergillus ochraceus*, *Fusarium graminearum*, and *Fusarium verticillioides* compared to the parent compound 7-hydroxy-4-methylcoumarin (Šarkanj et al., 2013). Additionally, it was noted that among six synthesized coumarin compounds, 7-hydroxy-4-methylcoumarin showed significant antimicrobial activity along with strong anti-larvicidal effects against *Culex quinquefasciatus* (Prabha and Nagarajan, 2016).

Previous molecular docking studies have identified several potential inhibitors for DdlA including quinic acid, 3-caffeoyl (Mansoori et al., 2022b), 2-(1,1-diallyl-but-3-enyl)-5-nonyl-phenol (Jaivel et al., 2017), and mucic acid (Dubey et al., 2023). A few other studies also reported the potential inhibitors of DdlA (Tahir et al., 2019; Barnes and Karatzas, 2020; Joshi et al., 2021a). Similarly, phytol, salicylic acid methyl ester (Mansoori et al., 2020), rosmarinic acid, piperanine, dihydropiperlonguminine, piperdardine, dihydrocurcumin and lonhumosides have been identified as potential inhibitors against the PDF of *Xoo* (Joshi et al., 2021a; Joshi et al., 2021b). Based on the molecular docking results obtained in the present study, together with previous reports, we proposed that 7-Hydroxy-4-methylcoumarin-3-acetic acid and other metabolites may contribute to the antibacterial activity against *Xoo* through the dual inhibition of DdlA and PDF.

### 3.7 Efficacy of extract on bacterial blight disease of rice

**FIGURE 10:**
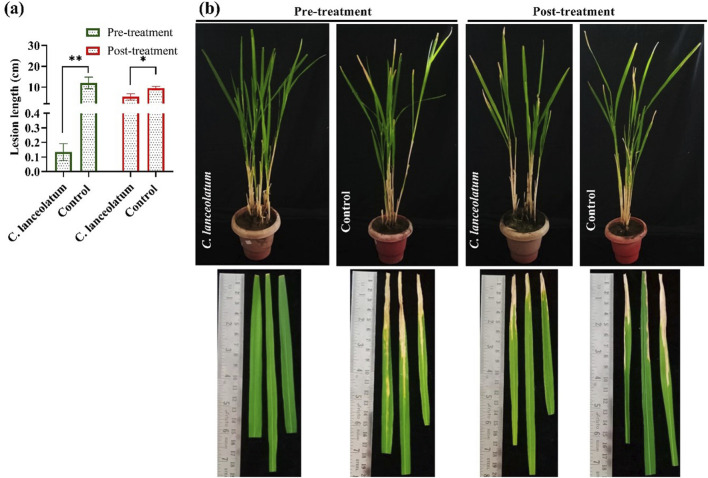
Disease protection effects of *C. lanceolatum* root extract on rice leaves against *Xoo*. Lesion score on rice leaves **(a)**. Rice leaf was pre and post treated with extractat12 h before and after *Xoo* infection using the leaf-clipping technique. Pictures were taken on 14th days after treatment **(b)**. Standard deviations of the means are indicated by vertical bars. Asterisks indicate statistically significant differences in lesion lengths (*p < 0.05; **p < 0.01; Student’s t-test) between control and extract-treated leaves.

*C. lanceolatum* extract was pre- and post-treated 12 h before and after *Xoo* inoculation. The lesion length measurement was done on the 14th day, which exhibited a minor *Xoo* lesion in pre-treated rice leaves of about 0.13 ± 0.05 cm. While lesion length was significantly (p < 0.01) higher in control rice (12 ± 2.78 cm). In the case of post-treatment, extract-treated rice leaves also observed significantly less lesion length (p < 0.05) than control (Figure 10). It was observed that pre-treatment of the extract was more effective than post-treatment. In pre-treatment, mechanical damage, rather than disease development, could be the cause of the little lesions detected at the point of the leaf clipping. Pre-treatment based protection against BB disease with plant extract before *Xoo* infection may be due to the priming effect, which prepares plants for rapid stress response (Ramirez-Prado et al., 2018). In agreement with the findings, the stimulation of priming implicit memory impacts the defense reaction to a subsequent pathogen attack, which involves the detection of a stimulus and the generation of a defense-related biochemical response (Aranega-Bou et al., 2014). A few reports also suggest that priming agents mimic pathogen interaction by acting as (endogenous or exogenous) elicitors, which plants sense to activate immunity. *Xoo* inoculation after 12 h of priming may result in pre-existing immunity in rice due to plant extract priming based on the above statement. The time gap between treatment and infection is only 12 h, which is enough for defense gene expression and other biochemical responses. After priming, the plant maintains its defense response for several days or sometimes up to the next-generation as evident from previous reports (Shasmita et al., 2019; Tiwari et al., 2022). Certain naturally occurring substances, including beta-aminobutyric acid, salicylic acid, jasmonic acid, pipecolic acid, chitosan, and hexanoic acid, provide actual evidence of priming-induced resistance in host plants (Llorens et al., 2016; Tiwari et al., 2022). However, post-treatment also exhibited higher and lower lesions than the pre-treated extract and control, respectively. Higher lesion length in post-treatment than pre-treatment can be linked to being a susceptible genotype of rice, which allowed less resistance due to more *Xoo* multiplication and their hijacking within 12 h. Secondary metabolites in plant defense have an intriguing function as inducers of defense resistance by regulating plant development and reducing pathogens. Secondary metabolites like phenolics, carotenoids, terpenes, and alkaloids aid plants in developing complex defense systems against various types of invading pathogens by enhancing metabolism for disease resistance (Abegaz and Kinfe, 2019; Anjali et al., 2023). Further, a previous report explained that molecules in the plant extract activate the defense-related signaling response in the host, such as MAP kinase, which most likely to inducesthe biochemicalresponse as usually SAR under salicylic acid regulation (Aranega-Bou et al., 2014; Jamiołkowska, 2020; Koziara et al., 2006). In contrast to justification, (Naz et al., 2018), has reported the induction of defence enzymes and pathogenesis-related protein expression in wheat enhanced by an exogenous foliar spray of botanical-chemical formulation against *Bipolaris sorokiniana*. Similarly, *Chorisia speciosa* extract have found to enhance systemic resistance to tomato root rot disease caused by *Rhizoctonia solani* (Behiry et al., 2022; Dubey et al., 2023 have particularly noticed the resistance in rice through pre-treatment of *Hedychium coronarium* flower extract against *Xoo*. *C. lanceolatum* has detected various metabolites which could explain the resistance inducer’s ability in rice. Finally, the results demonstrated that the plant *C. lanceolatum* extract generated considerably higher resistance than control. Thus, it can be useful to manage the BB of rice during *Xoo* exposure.

### 3.8 Effect on chlorophyll A fluorescence of rice

**FIGURE 11:**
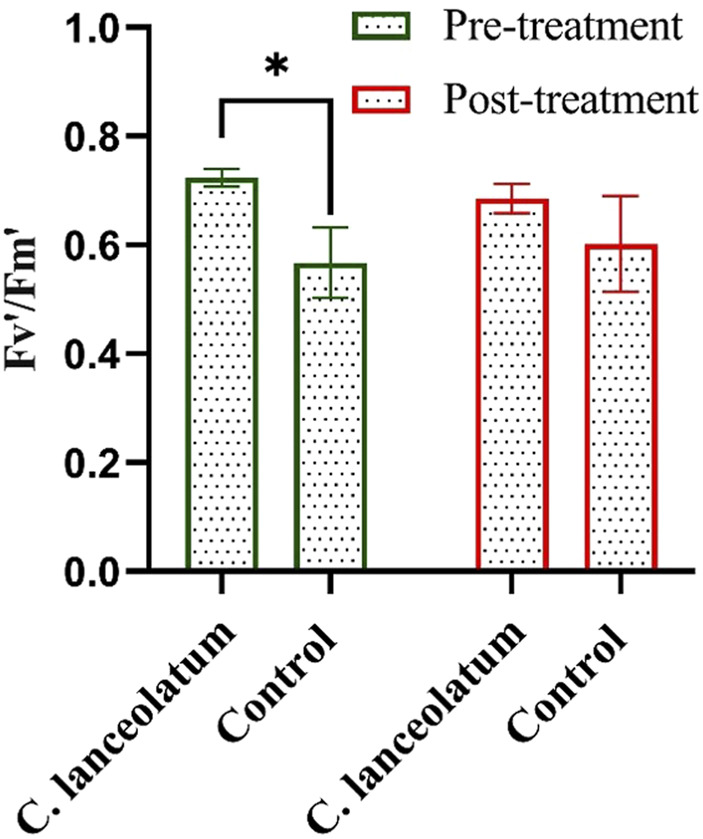
Chlorophyll A fluorescence measurement in rice leaves treated with *C. lanceolatum* root extract against bacterial blight disease. Each value is the average of four replicates (n = 3 ± SD). Symbols indicate the significance of difference according to Student’s t-test, * (p < 0.05) vs. control.

A pulse amplitude modulator (PAM) was used to evaluate chlorophyll A fluorescence on the 14th day after treatment. Assessing the photosynthetic efficiency of leaves on plants exposed to various abiotic and biotic stressors can be done by measuring the amount of chlorophyll fluorescence. Their values as Fv’/Fm’ were exhibited higher in both pre (0.72 ± 0.01) and post treatment (0.68 ± 0.02) than their individual control (Figure 11). Variations in the chlorophyll A fluorescence intensity within the chloroplasts indicate its functional state and offer crucial insights into the pigment systems' composition, excitation energy transfer, physical modifications in pigment-protein complexes, primary photochemistry and kinetics, and efficiency of photosystem II (PSII). Alteration of chlorophyll A fluorescence after treatment might be associated with BB infection which is further linked to reduced photosynthesis and carboxylation efficiency for loss of grain yield (Zhao et al., 2017). Several studies demonstrate that the presence of pathogens in plants frequently results in intricate modifications to their chlorophyll fluorescence, which may be connected to variations in the effectiveness of photosynthetic activities (Rolfe and Scholes, 2010; Scholes and Rolfe, 2009). Thus, the maintenance of the photosynthesis ability of rice explains the effectiveness of *C. lanceolatum* extract, or, in other terms, indicates no toxic effects of the extract on rice leaves.

## 4 Conclusion

The current investigation found that both methanol and aqueous extracts of *C. lanceolatum* root have better antioxidant and free radical scavenging properties. Quantitative investigation of phenol, flavonoid, tannin, and antioxidants confirms their slightly higher content in methanol extract compared to aqueous extract. The radical scavenging activity for DPPH and ABTS of methanol extracts was also found to be higher in a dose-dependent manner. The FTIR spectrum analysis revealed that both extracts had several functional groups, including OH, C-H, C=C, and C-N, which could be integral parts of different secondary metabolites. The GC-MS investigation for metabolite profiling demonstrated the presence of 59 compounds, with some of those having antimicrobial activity against pathogens as per the previous literature. Further, an antibacterial study showed promising inhibition with methanol extract against *Xoo*. Prediction of *in silico* antibacterial inhibition through the binding affinity of metabolites present in methanol extract confirmed the highest binding affinity of 7-hydroxy-4-methylcoumarin-3-acetic acid with DdlA and PDF proteins of *Xoo*. Further, in the bacterial blight protection assay, pre- and post-treatment with aqueous extract gave evidence to play an important role as inducers of resistance against BB of rice caused by *Xoo*. The tested extracts significantly decreased the disease incidence of *Xoo*, especially in pre-treatment, as represented by less lesion length. Higher chlorophyll A fluorescence in extract-treated rice leaves under both pre- and post-treatment was observed in comparison to their respective controls, which suggests PS ІІ protection from *Xoo.* Finally, it was assumed that the *C. lanceolatum* root extract was able to play a versatile role as an antioxidant, antimicrobial, and resistance inducer in rice against BB disease. Further investigations should be conducted to identify the expression of defense marker genes in rice during treatments to have a thorough understanding of the defense pathway.

## Data Availability

The datasets presented in this study can be found in online repositories. The names of the repository/repositories and accession number(s) can be found in the article/Supplementary Material.
