# Supplementary material for: Integrated metabolomics and bioactivity analysis of Cynoglossum lanceolatum root extract as a natural inducer of resistance to rice bacterial blight
Source: Front Genet. 2026 Apr 22;17:1775858. doi: 10.3389/fgene.2026.1775858 (PMC13143228; doi:10.3389/fgene.2026.1775858)
Supplement: Supplementary file 1 [file Supplementaryfile1.docx]

***Cynoglossum lanceolatum* root extract treatment induces resistance to bacterial blight of rice with greater antioxidant and antibacterial activity**

**Aadil Mansoori^1^, Priyanka Prasad^1^, Madan Mohan^1^, Subha Narayan Das^1^, Kapil Sharma^2^, Anirudh Kumar^1,3,*^**

^1^Department of Botany, Indira Gandhi National Tribal University (IGNTU), Amarkantak, M.P., India

^2^Department of Plant Sciences, University of Hyderabad, Hyderabad-,500046 India.

^3^Department of Botany, Central Tribal University of AP, Vizianagaram-535003, A.P., India

***Corresponding author**

Anirudh Kumar

Department of Botany

Central Tribal University of AP, Vizianagaram (AP)-535003, India. Email: anirudh@ctuap.ac.in


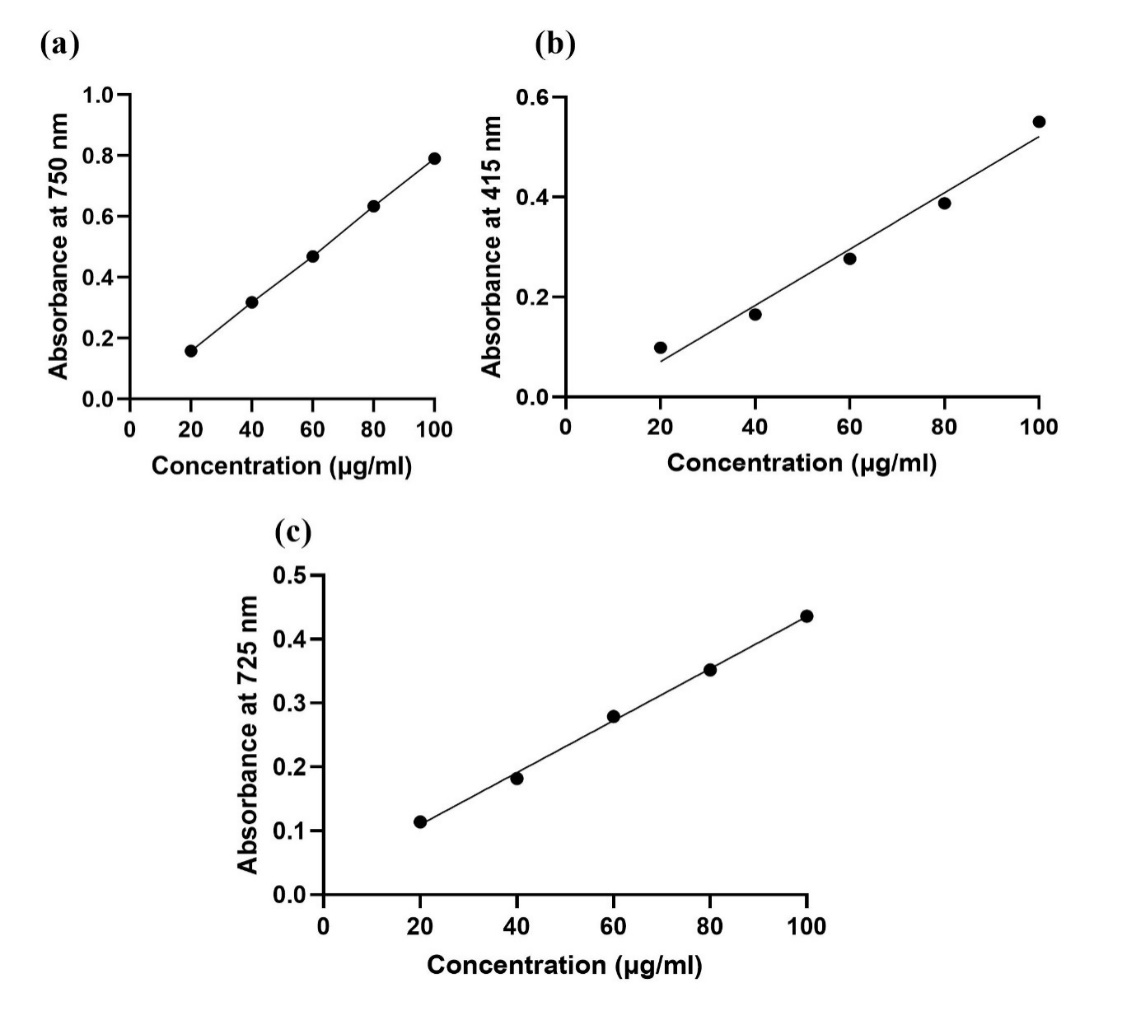


**Supplementary Fig. 1** Calibration curve of gallic acid (a), quercetin (b) and tannic acid (c).


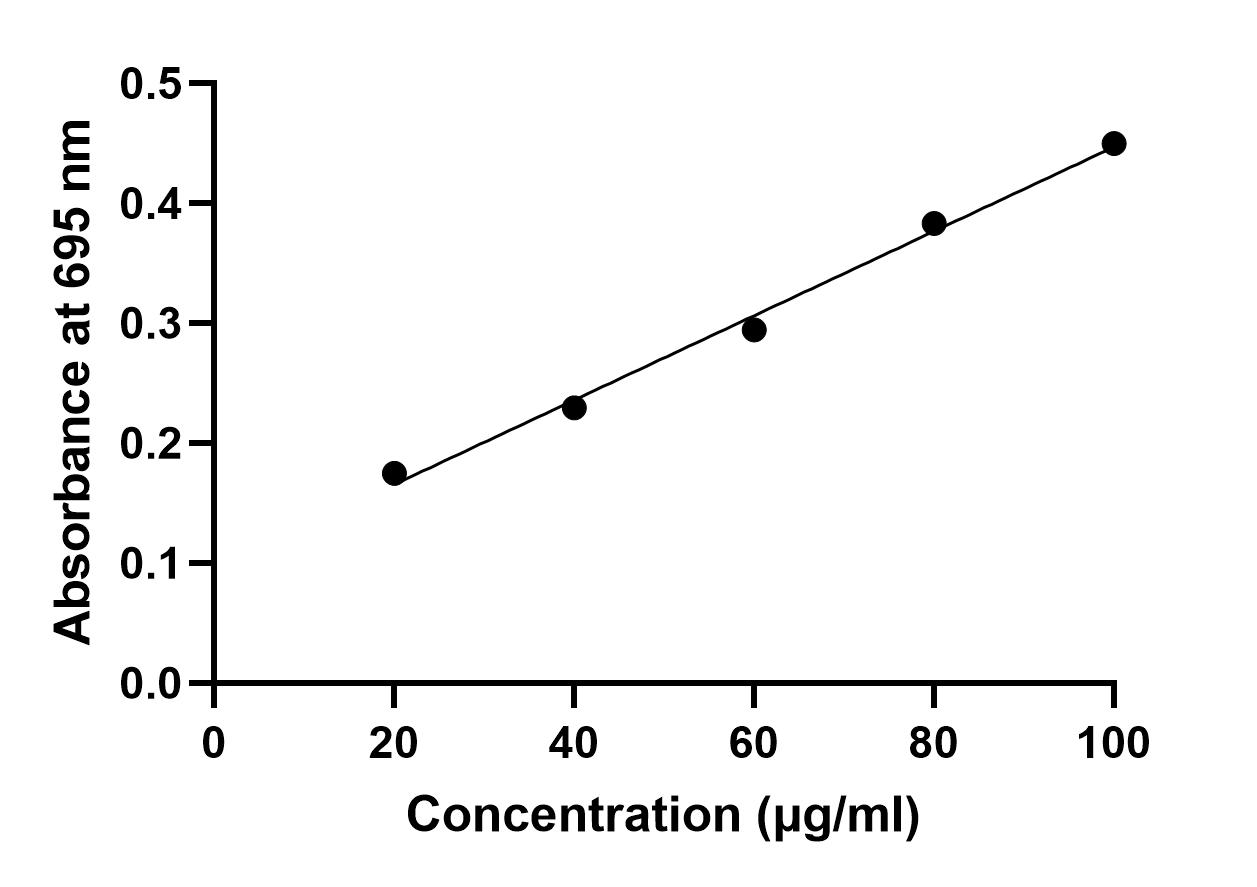


**Supplementary Fig. 2** Calibration curve of ascorbic acid.

Supplementary Table 1: Previously reported antimicrobial metabolites identified in methanol extract of *C. lanceolatum* root.

| **Compound** | **Compound Nature** | **Biological activity** | **Reference** |
| --- | --- | --- | --- |
| Pyruvic acid | Organic acid | Individual or synergistic antibacterial effects with oregano essential oil against Salmonella *typhimurium*. | (Mohan & Purohit, 2020) |
| Glycolic acid | Organic acid | pH dependent antibacterial activity against *Cutibacterium acnes* | (Valle-González et al., 2020) |
| 3-Hydroxypropionic acid | Organic acid | Antibacterial activity against *Staphylococcus aureus* and *Salmonella typhi* | (Sebastianes et al., 2012) |
| Phosphoric acid | Organic acid | Antibacterial activity against *Candida albicans, Staphylococcus aureus* | (Prado et al., 2015) |
| Succinic acid | Organic acid | Inhibitory effects on Staphylococcus aureus and Pseudomonas fluorescens through cell membrane damage and and intracellular content leakage. | (Huang et al., 2022) |
| Fumaric acid | Organic acid | Antibacterial activity against [*Listeria monocytogenes*](https://www.sciencedirect.com/topics/immunology-and-microbiology/listeria-monocytogenes) biofilm removal. | (Barnes & Karatzas, 2020) |
| Linoleic acid | Fatty acid | Synergistic antibacterial effects with oleic acid against *Staphylococcus aureus* and *Micrococcus kristinae* | (Dilika et al., 2000) |
| Stearic acid | Fatty acid | Inhibitory effects on *Bacillus cereus* and *Bacillus subtilis* and fast-acid bacterium *Mycobacterium fortuitum*. | (Dantas Da Silva et al., 2002) |
| Echinatine | Alkaloid | Synergistic anti-quorum effects with antibiotics (colistin B and colistin E) against *Escherichia coli*. | (Bai et al., 2022) |
| Caffeic acid | Phenolics | Antibacterial effects on *Staphylococcus aureus.* | (Ma et al., 2018) |

Supplementary Table 2: Hydrogen bond and hydrophobic interaction of top six compounds with DdlA and PDF of *Xoo*.

| **Complex** | **Binding**  **Affinity**  **(Kcal/mol)** | **Hydrogen**  **Bond** | | | **Hydrophobic** | | |
| --- | --- | --- | --- | --- | --- | --- | --- |
|  |  | **Bonding**  **Type** | **Protein** | **Distance**  **(Å)** | **Bonding**  **Type** | **Protein** | **Distance**  **(Å)** |
|  |  |  | **Interacting**  **Amino acid** |  |  | **Interacting**  **Amino acid** |  |
| 7-Hydroxy-4-methylcoumarin-3-acetic acid-DdlA | -7.2 | Conventional | LysA:13,  GlyA:108 | 2.00  2.41 | Alkyl | Ile:143,  Val:106  Leu:110  Leu:116 | 5.07  3.93  5.35  3.61 |
|  |  |  |  |  | Pi-alkyl | Leu:110  Leu:116  Leu:119 | 4.67  5.34  5.30 |
|  |  |  |  |  | Pi-Sigma | Leu:110 | 3.88 |
| 3,4-Dihydroxyphenylacetic acid-DdlA | -5.9 | Conventional | His:107  Gly:108 | 2.70  2.75 | Pi-alkyl | Val:106  Leu:110  Leu:116 | 5.12  4.79  5.05 |
|  |  |  |  |  | Pi-sigma | Ile:43 | 3.89 |
|  |  |  |  |  |  |  |  |
| Dihydroxyphenylalanine-DdlA | -5.9 |  |  |  | Pi-alkyl | Val:106  Leu:110  Leu:116 | 5.29  4.79  4.91 |
|  |  |  |  |  | Pi-sigma | Ile:43 | 3.99 |
| 7-Hydroxy-4-methylcoumarin-3-acetic acid-PDF | -6.8 | Conventional | Tyr:69  Glu:93  Trp:96  Arg:137 | 2.47  2.21  2.94  4.54 | Alkyl | Ala:72 (2)  Pro:73 | 5.09, 4.05  4.89 |
|  |  | Carbon hydrogen | Gly:95 (2) | 3.34,  3.70 | Pi-alkyl | Phe:134 | 4.76 |
|  | | | | | Pi-Pi T-shaped | Tyr:69 | 5.22 |
| 4-N-Methylaminobutyricacid-PDF | -6.6 | Conventional | Tyr:69  Glu:97  Gly:98 | 2.65  2.61  2.37 |  |  |  |
|  |  |  |  |  |  |  |  |
| Caffeic acid-PDF | -5.9 | Conventional | Arg:68 (2)  Cys:99  Ile:102  Gly:104 | 2.78, 2.89  2.16  2.52  2.18 | Pi-alkyl | Arg:68  Arg:106 | 5.09  4.22 |
